# Genetic Variation in miR-27a Is Associated with Fluoropyrimidine-Associated Toxicity in Patients with Dihydropyrimidine Dehydrogenase Variants after Genotype-Guided Dose Reduction

**DOI:** 10.3390/ijms241713284

**Published:** 2023-08-27

**Authors:** Samantha Medwid, Theodore J. Wigle, Cameron Ross, Richard B. Kim

**Affiliations:** 1 Department of Medicine, University of Western Ontario, London, ON N6A 3K7, Canada; samantha.medwid@lhsc.on.ca (S.M.); theo.wigle@lhsc.on.ca (T.J.W.); cameron.ross@lhsc.on.ca (C.R.); 2 London Health Sciences Centre, London, ON N6A 5A5, Canada; 3 Lawson Health Research Institute, London, ON N6C 2R5, Canada

**Keywords:** *DPYD*, fluoropyrimidines, 5-fluorouracil, miRNA, miR-27A, pharmacogenomics

## Abstract

Dihydropyrimidine dehydrogenase (*DPYD*) is the rate-limiting enzyme involved in the metabolism of fluoropyrimidine-based chemotherapy. However, single-nucleotide variants (SNVs) in *DPYD* only partially explain fluoropyrimidine-induced toxicity. The expression of *DPYD* has previously been shown to be regulated by microRNA-27a (miR-27a) and a common miR-27a SNV (rs895819) has been associated with an increased risk of toxicity in patients harboring a *DPYD* variant who received standard fluoropyrimidine dosing. We investigated if the miR-27a rs895819 SNV was associated with toxicity in *DPYD* wildtype patients and carriers of *DPYD* variants who received a reduced dose. The regulation of *DPYD* using miR-27a was investigated in HepG2 cells utilizing a miR-27a mimic. miR-27a overexpression decreased *DPYD* mRNA expression compared to control cells (*p* < 0.0001). In a cohort of patients that received pre-emptive *DPYD* genotyping, 45 patients had a *DPYD* variant and 180 were wildtype. Patients heterozygous for rs895819 had an increased risk of toxicity, which was seen in both patients who were wildtype for *DPYD* variants (OR (95%CI) = 1.99 (1.00–3.99)) and *DPYD* variant carriers (OR (95%CI) = 8.10 (1.16–86.21)). Therefore, miR-27a rs895819 may be a clinically relevant predictor of fluoropyrimidine-associated toxicities. Furthermore, toxicity was more profound in *DPYD* variant carriers, even after *DPYD* genotype-guided dose reduction. This suggests that patients may benefit from miR-27a genotyping to guide fluoropyrimidine dosing.

## 1. Introduction

Fluoropyrimidines, such as 5-fluorouracil or the oral prodrug capecitabine, are widely used in the treatment of solid tumors. Over 30% of patients develop severe toxicity from fluoropyrimidine therapy [1]. Dihydropyrimidine dehydrogenase (DPD, gene name *DPYD*) is the rate-limiting step in the metabolism of 5-fluororuracil (5-FU) into its inactive metabolite dihydrofluorouracil (DHFU). Therefore, deficiency in DPD leads to increased systemic levels of fluoropyrimidines, increasing the risk of toxicity. [1]. Currently, genotype-guided dosing is recommended for patients with the *DPYD* single-nucleotide variations (SNVs), c.1905+1G>A (rs3918290), c.2846A>T (rs67376798), c.1679T>G (rs55886062), and c.1236G>A (rs56038477; in lieu of the linked c.1129-5923C>G SNV) to reduce the risk of toxicity [2]. However, adjusting for these variants only accounts for 20–30% of the toxicity observed and does not fully explain the variability in DPD activity. Therefore, further investigation into other regulatory mechanisms of DPD activity is needed to optimize fluoropyrimidine dosing.

MicroRNAs (miRNA) are single-stranded and short RNAs, 20–22 nucleotides in length, that are involved in post-transcriptional gene regulation [3,4]. miRNA binds and targets the 3′ and 5′ untranslated regions of genes, resulting in the inhibition of gene translation [4]. Numerous miRNAs have been investigated due to their potential role as cancer biomarkers and association with cancer occurrence and prognosis [5,6]. miRNAs have been reported as both oncogenes and tumor suppressors, and their function depends on their mRNA targets and the cell type [5,7].

Not surprisingly, SNVs in miRNAs have been reported to alter the stability, and therefore their functionality, on target genes. Furthermore, SNVs in miRNAs have been explored as risk factors for cancer occurrence and chemotherapy resistance. One such SNV in miR-27a, rs895819, has been extensively studied in relation to the risk and prognosis of various cancers; however, results have been unclear. For example, in two Caucasian cohorts, there was no association with colorectal cancer risk [8,9]; however, multiple studies in Asian cohorts have reported an increased risk of cancer occurrence [10,11,12,13]. Several meta-analysts concluded that the presence of the rs895819 SNV is associated with colorectal cancer, with most studies finding this to only be significant in patients’ homozygous for the variant allele and not carriers [14,15,16,17,18,19,20,21]. However, in contrast, three meta-analyses found no association of the rs895819 SNV with colorectal cancer [22,23,24]. Additionally, recent meta-analyses have shown miR-27a rs895819 SNVs to be associated with an increased risk of gastric cancer, a decreased risk of breast cancer, and no effect on lung or esophageal cancer [17,25].

Regulation of *DPYD* by various miRNAs has been previously shown [26]. For example, miR-27a, through direct binding to the 3′ untranslated region of *DPYD*, leads to decreased DPD activity, mRNA levels, and protein expression [27,28]. Furthermore, the miR-27a rs895819 SNV is predicted to increase miR-27a stability, thus resulting in increased miR-27a expression in lymphoblastoid cell lines and decreased DPD activity in carriers of the rs895819 SNV [27]. Accordingly, a small study of 64 colorectal cancer patients showed an association between carrying the miR-27a rs895819 SNV and severe toxicity in patients receiving fluoropyrimidines, oxaliplatin, and irinotecan [29]. However, this association did not remain significant when other genotypes, including *DPYD* c.496A>G, c.1896T>C, and UGT1A1*28, were accounted for [29]. However, two large studies demonstrated that patients with miR-27a rs895819, who also harbored a *DPYD* risk variant (c.1905+1G>A, c.2846A>T, c.1679T>G, or c.1236G>A), and did not receive genotype-guided dosing, had an increased risk of fluoropyrimidine-related toxicity. However, in patients who are wildtype for these *DPYD* SNVs, there were conflicting results, with one study reporting that miR-27a rs895819 SNV decreased the risk of toxicity [30] and another reporting a modest increased risk of toxicity [31]. Therefore, we sought to investigate if the miR-27a rs895819 SNV was associated with fluoropyrimidine toxicity in patients receiving *DPYD* genotype-guided standard as well as a reduced fluoropyrimidine dose.

## 2. Results

### 2.1. miR-27a-3p Regulation of DPYD Expression

**Figure 1:**
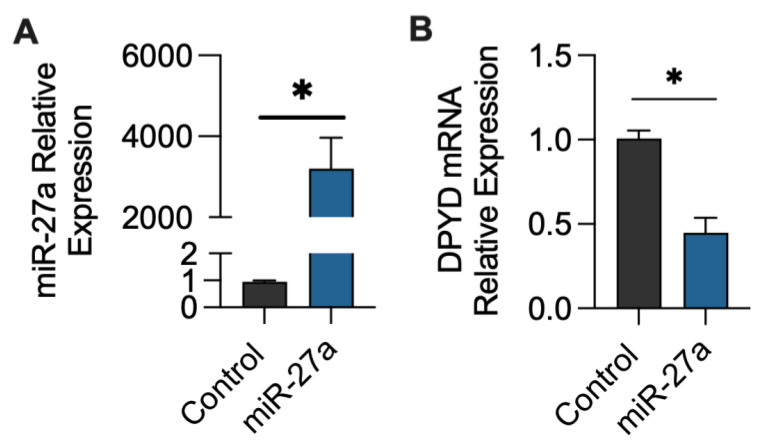
miR-27a overexpression inhibited *DPYD* mRNA expression. The relative expression of (**A**) miR-27a and (**B**) *DPYD* mRNA in HepG2 cells after 48 h of exposure to a miR-27a mimic or control (transfection reagents alone). Relative expression was calculated using the ΔΔCT method with U6 siRNA and *GAPDH* as housekeeping genes for miR-27a and *DPYD*, respectively. Data are shown as mean ± SEM, N = 4, * *p* < 0.05.

miR-27a-3p expression was measured in HepG2 liver cells after transfection with a miR-27a-3p mimic. The expression of miR-27a-3p was dramatically increased in cells transfected with miR-27a-3p compared to control cells (3200%, *p* < 0.05; Figure 1A). Furthermore, *DPYD* mRNA expression was decreased by 56% in cells transfected with a miR-27a mimic compared to control cells (*p* < 0.05; Figure 1B).

### 2.2. Study Population

Among the 225 patients, the median age was 62, 48% were male, and 53% had colorectal cancer. *DPYD* variants were previously found in 45 patients, with 18, 9, and 18 patients being carriers of c.2846A>T, c.1905+1G>A, and c.1236G>A, respectively. The minor allelic frequency (MAF) of miR-27a rs895819 and rs11671784 were 0.36 and 0.016, respectively. Baseline demographics are shown in Table 1. The initial dose of fluoropyrimidines were 52% and 90% of the ideal dose in patients with *DPYD* variants and without *DPYD* variants, respectively. Similarly, the average fluoropyrimidine dose intensity rates were 54% and 87% in patients with and without *DPYD* variants, respectively (Table 2). Patients carrying *DPYD* variants who had received a fluoropyrimidine dose adjustment were not at a greater risk of fluoropyrimidine-associated toxicity compared to *DPYD* wildtype patient during cycles 1 and 2 (OR (95% CI) = 0.53 (0.20–1.21)) or during the total treatment period (OR (95% CI) = 0.67 (0.31–1.36)) (Table S1).

### 2.3. Association of miR-27a SNVs and Fluoropyrimidine-Associated Toxicity in the Total Patient Population

In the overall sample (*DPYD* wildtype and variant carriers), patients with one variant allele of miR-27a rs895819 were at an increased risk of fluoropyrimidine-associated toxicity during their total treatment period (OR (95% CI) = 2.38 (1.26–4.57)), while patients with two variant alleles were not at an increased risk (OR (95%) = 1.49 (0.63–3.43)) (Table 3). Similarly, during chemotherapy cycles 1 and 2, patients with one variant allele of miR-27a were at an increased risk of fluoropyrimidine-associated toxicity (OR (95% CI) = 2.29 (1.14–4.71)), while no association was found with two alleles (OR (95% CI) = 1.05 (0.37–2.75)) (Table 4). Within the overall population, patients with one variant allele of miR-27a rs895819 had increased severe gastrointestinal, myelosuppression, cardiac, and hand–foot syndrome toxicities compared to wildtype patients. Additionally, they had higher discontinuation of fluoropyrimidine treatment due to related toxicities (23%) compared to wildtype patients (16%) (Table 5 and Table 6). miR27-a rs11671784 was not associated with an increase in fluoropyrimidine-associated toxicity in the total population (OR (95%) = 0.33 (0.02–2.06)) (Table 3).

### 2.4. Association of miR-27a SNVs and Fluoropyrimidine-Associated Toxicity in Patients’ Wildtype for DPYD 

Previous studies have suggested that miR-27a SNVs were only associated with fluoropyrimidine-related toxicities in patients also harboring a *DPYD* variant and not wildtype patients [30]. As such, we looked at the association with miR-27a SNVs in these separate groups. An increased risk of fluoropyrimidine-associated toxicity during total treatment was seen in *DPYD* wildtype patients who were heterozygous for miR-27a rs895819 (OR (95% CI) = 1.99 (1.00–3.99)), while no difference was seen in patients homozygous for miR-27a rs895819 (OR (95% CI) = 1.44 (0.58–3.49)) during the total treatment period (Table 3). Similarly, in patients who were wild-type for *DPYD*, an association with fluoropyrimidine-related toxicities during cycles 1 and 2 was found in patients heterozygous for miR-27a rs895819 (OR (95% CI) = 2.30 (1.09–4.97)), but not those homozygous for the variant (OR (95%) = 1.21 (0.41–3.27)) (Table 4). Furthermore, patients heterozygous for miR-27a rs895819 saw an increase in gastrointestinal and myelosuppression severe toxicities compared to wildtype patients, as well as higher rates of treatment discontinuation (Table 5 and Table 6).

### 2.5. Association of miR-27a SNVs and Fluoropyrimidine-Associated Toxicity in DPYD Variant Carriers

*DPYD* variant carriers (*DPYD* c.1905+1G>A, c.2846A>T, c.1679T>G, and c.1236G>A) who were given a dose adjustment were at an 8.10 times higher risk of fluoropyrimidine-associated toxicity with one allele of miR-27a rs895819 (OR (95%) = 8.10 (1.16–86.21)) but not two alleles (OR (CI 95%) = 3.87 (0.12–90.67)) (Table 3). Due to the small sample size of this study, we were unable to determine the risk of toxicity during cycles 1 and 2 (Table 4). *DPYD* variant carriers that were heterozygous for miR-27a rs895819 had an increase in gastrointestinal and hand–foot syndrome severe toxicity during fluoropyrimidine treatment. Additionally, *DPYD* variant carriers that also had a miR-27a rs895819 variant had an increase in the discontinuation of fluoropyrimidine treatment due to related toxicities (32%) compared to patients with a *DPYD* variant alone (20%) (Table 5 and Table 6).

## 3. Discussion

The predictive role of deleterious *DPYD* on fluoropyrimidine toxicity has been well established; however, known *DPYD* SNVs are unable to account for the majority of fluoropyrimidine-associated toxicity. In this study, we were able to show that miR-27a can regulate *DPYD* expression in vitro. As well as in vivo, the common rs895819 SNV was predictive of fluoropyrimidine-associated toxicity during genotype-guided dose reduction. Importantly, we have shown for the first time that patients harboring a *DPYD* variant (*DPYD* c.1905+1G>A, c.2846A>T, c.1679T>G, and c.1236G>A) that received a genotype-guided dose reduction were still at risk for increased toxicity if they were also a carrier of miR-27a rs895819 SNV.

miR-27a has previously been shown to directly regulate *DPYD* expression in vitro [27,28]. We have confirmed that overexpression of miR-27a inhibited *DPYD* mRNA expression in HepG2 cells. Similarly, previous studies demonstrated that *DPYD* protein and mRNA expression were inhibited after treatment with a miR-27a mimic [27,28]. miR-27a is predicted to bind to the 3′UTR region of *DPYD* and directly affect its expression [27]. Therefore, the role of miR-27a in the regulation of *DPYD* may have a significant effect on the expression and total activity of DPD, and may subsequently influence fluoropyrimidine metabolism and toxicity. Furthermore, using the miRDB database (http://www.mirdb.org (accessed on 21 August 2023)) for miRNA-mRNA predictions, no other enzymes in the fluoropyrimidine metabolism pathway were predicted to be targets of hsa-miR27A-3p, other than *DPYD*. However, the clinical relevance of miR-27a plasma levels as a predictor of *DPYD*-induced fluoropyrimidine toxicity remains to be known.

In this study, we show that the common miR-27a rs895819 SNV (MAF = 0.36) was associated with increased fluoropyrimidine toxicity in *DPYD* wildtype and variant carriers even after appropriate dosing among variant carriers. Previous studies of *DPYD* variant carriers, not pre-emptively dose-adjusted, show an increased risk of fluoropyrimidine-toxicity between 4.6–7.4 times higher in carriers of the rs895819 SNV than wildtype patients [29,30,31]. To the best of our knowledge, the current study is the first to demonstrate an association with the rs895819 genotype and the increased risk of fluoropyrimidine toxicity among *DPYD* variant carriers who had received appropriate genotype-guided reduction (OR (95%) = 8.10 (1.16–86.21)). *DPYD* variant carriers in this study had, on average, 48% and 46% reduction in initial and average dose over the treatment period, respectively, resulting in similar fluoropyrimidine toxicity rates between *DPYD* variant carriers and wildtype patients (27% versus 35%, respectively). Therefore, the pre-emptive genotype-guided dose reduction in patients with *DPYD* variants is not a sufficient reduction if patients also harbor the miR-27a rs895819 SNV. Thus, an additional dose adjustment on top of the current *DPYD* recommendations may be necessary for 30% of *DPYD* carriers also harboring the miR-27a rs895819 SNV.

In patients with no known *DPYD* variants, we showed an association of fluoropyrimidine toxicity in carriers of rs895819 (OR (95% CI) = 1.99 (1.00–3.99)). This is similar to Meulendijks et al. [31] who showed a small but significant association of toxicity in patients carrying the miR-27a rs895819 SNV (OR (95%) = 1.5 (1.00–2.14)), in contrast to the decreased risk of toxicity shown by Amstutz et al. [30] (OR (95%) 0.73 (0.56–0.97)). Therefore, this demonstrates that the effect of miR-27a is not exclusive to only *DPYD* variant carriers, and the consideration of miR-27a to fluoropyrimidine therapy may be beneficial to all patients. We note however that we did not account for additional *DPYD* variants, including rare variants or c.1601G>A in our *DPYD* “wildtype” populations. Interestingly, Meulendijks et al. found that *DPYD* c.1601G>A, in combination with miR-27a SNVs (rs895819 and/or rs11671784), increased their risk of severe fluoropyrimidine toxicity [31]. Thus, some of the effects seen in our *DPYD* wildtype patients may be partly due to the presence of other *DPYD* variants.

The miR-27a rs11671784 SNV is much less common than rs895819, with a minor allele frequency of 0.016 in our cohort. In this study, we found no association with fluoropyrimidine toxicity in the carriers of rs11671784 SNV. However, due to the low frequency, we only found seven carriers of the gene and we were unable to stratify further into *DPYD* wildtype and variant carriers. Similarly, previous reports showed no association of rs11671784 with fluoropyrimidine toxicity [30,31]. Furthermore, Amstutz et al. reported sex differences in the effect of rs11671784 SNV, suggesting that it is protective in female patients only and not males [30]. Unfortunately, due to the small sample size, we were unable to investigate this in our cohort. Previous studies have suggested that the rs11671784 SNV results in a decrease in miR-27a expression [32,33]; thus, this could lead to an increase in DPD expression and activity. Interestingly, these papers report an allele frequency of rs11671784 SNV to be much higher in their Chinese cohorts compared to ours and previous Caucasian cohorts [30,31]. Further studies on the effect of the rs11671784 SNV on the regulation of *DPYD* and any potential sex or ethnicity differences are needed.

Both rs895819 and rs11671784 are located in the terminal loop of the pre-microRNA, four nucleotides apart. As these SNVs are present in the miR-27a hairpin loop, they do not interfere with the binding site of the mature miR-27a to *DPYD* [27]. The presence of the rs11671784 SNV has been previously shown to decrease the expression of miR-27a expression [33,34]. However, the mechanism of the rs895819 SNV and its effect on its expression is unknown. Previous studies suggested that the rs895819 SNV alters the stability and/or processing of miR-27a; however, these results are conflicting, with some studies showing increased miR-27a expression [13,34], others showing decreased miR-27a expression [27,35,36], and one showing no effect of the rs895819 SNV on miR-27a expression [37]. Similarly, conflicting studies have shown a decrease, an increase, and no association between carriers of the rs895819 SNV and the risk of various cancers [16,17,18,25,38]. Interestingly, differences in the risk of cancer susceptibility with the rs895819 SNV are suggested due to ethnicity differences, with Asian carriers of rs895819 having an increased risk, but there has been no association seen in Caucasian populations [16,18,38]. Similarity ethnicity-specific effects of the rs895819 SNV are seen in other conditions, such as recurrent spontaneous abortions [39] and type II diabetes [40]. We were unable to examine ethnicity differences relating to the effects of the rs895819 SNV in our study due to >95% of our patients being of Caucasian descent.

## 4. Materials and Methods

### 4.1. Cell Culture

Human embryonic kidney type T (HEK293T) cells were obtained from American Type Culture Collection (Manassas, VA, USA). Cells were cultured at 37 °C, with 5% CO_2_, in Dulbecco’s Modified Eagle Medium (DMEM; Thermo Scientific, Waltham, MA, USA) containing 10% fetal bovine serum (FBS), 100 U/mL penicillin, and 2 mM L-glutamine (Invitrogen, Waltham, MA, USA).

### 4.2. miR-27a mimic Transfection

A miR-27a-3p mimic (Thermo Fisher, Waltham, MA, USA, Assay Id MC10939) was transfected into HepG2 24 h after plating. Reverse transfection was performed by adding RNAiMAX lipofectamine (Thermo Fisher) and the miR-27a-3p mimic (final concentration 40 nM) to 24-well plates for 15 min before the addition of cells. After 48 h, the cells were harvested for RNA extraction.

### 4.3. Real-Time RT-PCR

Total RNA was extracted from HEK293T cells 48 h after transfection with a miR-27a mimic using the TRIZOL method, as previously described [41]. For miRNA levels, reverse transcription was performed with the TaqMan MicroRNA Reverse Transcription Kit, according to manufacturer’s instructions, followed by real-time PCR using TaqMan assays (miR-27a: assay ID 000408 and U6 SNRNA assay ID 001973). To determine mRNA levels of *DPYD* and GAPDH, cDNA was created using the iScript reverse transcription kit (BioRAD). Real-time PCR was performed using SYBR green reagents and specific primers (*DPYD* forward: GGTGGTGATGTCGTTGGTTT, *DPYD* reverse: GCAGAAACGGAAGCTCCATA, GAPDH forward: ACCACAGTCCATGCCATCAC, GAPDH reverse: TCCACCACCCTGTTGCTGTA). All real-time PCR was performed using a Viia7 Real-Time PCR machine (ThermoFisher). Relative expression was calculated using the ΔΔCT method, with U6 siRNA and *GAPDH* used as housekeeping genes for miR-27a and *DPYD*, respectively.

### 4.4. Study Design

A subset of previously published patients who were pre-emptively genotyped for *DPYD* variants c.1905+1G>A, c.2846A>T, c.1679T>G, and c.1236G>A prior to dosing with fluoropyrimidines were used [42]. Fluoropyrimidine-guided dose reduction was performed according to previously published methods using TaqMan genotyping [42]. Severe adverse events (AEs) included grade ≥3 toxicities, according to the National Cancer Institutes’ Common Terminology Criteria for Adverse Events (CTCAE) (version 5.0). The study was approved by the Institutional Review Board at Western University. Written informed consent was obtained from all individuals participants in the study.

### 4.5. Genotyping and Sequencing of miR-27a

Whole blood samples were collected, and DNA was extracted using a MagNA Pure Compact instrument (Roche). Genotyping of miR-27a variants rs895819 and rs11671784 was completed using Sanger sequencing due to the proximity of variants [43]. PCR was performed for miR-27a using primers 5′-GTCCCCAAATCTCATTACCTCCTT-3′ (forward) and 5′-GGTCTGATTCTGAGTCCTCATCTC-3′ (reverse) with AmpliTaq Gold DNA Polymerase (Thermo Scientific), and an annealing temperature of 58 °C for 35 cycles. Sanger sequencing was performed with the same primers as listed above. A representative image of Sanger sequencing results showing a homozygous wildtype, a homozygous variant, and a heterozygote for miR-27a rs895819 is shown in Figure S1.

### 4.6. Statistical Analysis

Statistical analysis was performed using GraphPad Prism 9 with Student’s t-tests used for the in vitro analysis. For the association of miR-27a SNVs with toxicities, a logistic regression model adjusting for age, sex, treatment type (5-fluorouracil or capecitabine), and *DPYD* genotype (total population and *DPYD* variant carrier analyses only) was used. Odds ratios and 95% confidence intervals were shown compared to the wildtype genotype as the reference group. A *p*-value of less than 0.05 was considered statically significant.

## 5. Conclusions

In summary, we demonstrate new findings to support an important role of the miR-27a rs895819 SNV as a predictor of toxicity, even among *DPYD* variant carriers who receive appropriate fluoropyrimidine dose reduction. Furthermore, we report that *DPYD* wildtype patients are at an increased risk of fluoropyrimidine toxicity if they carry rs895819. Therefore, our findings suggest that, in addition to *DPYD*, patients may benefit from miR-27a genotyping to guide fluoropyrimidine dosing; thus, the clinical impact of miR-27a expression, regulation, and SNVs warrant additional studies.

## Figures and Tables

**Table 1:** Baseline demographics.

	Total Population (N = 225)	Patients with *DPYD* Variants ^1^ (N = 45)	Patients with No *DPYD* Variants ^1^ (N = 180)
**Age (range)**	62 (33–89)	63 (34–86)	62 (33–89)
**Sex, N (%)**			
Male	107 (48)	21 (47)	86 (48)
Female	118 (52)	24 (53)	94 (52)
**Tumor Site, N (%)**			
Colorectal	119 (53)	23 (51)	96 (53)
Gastric and esophagus	35 16)	7 (16)	28 (16)
Pancreas	24 (11)	6 (13)	18 (10)
Breast	12 (5)	3 (7)	9 (5)
Head and neck	9 (4)	2 (4)	7 (4)
Anal	8 (4)	1 (2)	7 (4)
Other ^2^	18 (8)	3 (7)	15 (8)
***DPYD* Genotype, N (%)**			
Wildtype	180 (80)	0 (0)	180 (100)
c.2846A>T	18 (8)	18 (40)	0 (0)
c.1905+1G>A	9 (4)	9 (20)	0 (0)
c.1679T>G	0 (0)	0 (0)	0 (0)
c.1236G>A	18 (8)	18 (40)	0 (0)
**miR-27a rs895819, N (%)**			
A/A	100 (44)	20 (44)	80 (44)
A/G	88 (39)	19 (42)	69 (38)
G/G	37 (16)	6 (13)	31 (17)
**miR-27a rs11671784, N (%)**			
C/C	218 (97)	44 (98)	174 (97)
C/T	7 (3)	1 (2)	6 (3)
T/T	0 (0)	0 (0)	0 (0)

^1^ *DPYD* variants include c.2846A>T, c.1905+1G>A, c.1679T>G, and c.1236G>A; ^2^ includes appendix and small bowel, genitourinary, hepatobiliary, and unknown primary sites.

**Table 2:** Chemotherapy characteristics.

	Total Population (N = 225)	Patients with *DPYD* Variants ^1^ (N = 45)	Patients with No *DPYD* Variants ^1^ (N = 180)
**Regimen, N (%)**			
Capecitabine monotherapy ^2^	39 (17)	10 (22)	29 (16)
Capecitabine with radiation	36 (16)	8 (18)	28 (16)
Capecitabine with oxaliplatin	21 (9)	2 (4)	19 (11)
Capecitabine with other agents ^3^	16 (7)	3 (7)	13 (7)
FOLFOX ^2^	42 (19)	7 (16)	35 (19)
FOLFIRI/FOLFIRINOX	22 (10)	7 (16)	15 (8)
5-FU with cisplatin–carboplatin	26 (12)	4 (9)	22 (12)
5-FU with other agents ^4^	23 (10)	4 (9)	19 (11)
**BSA, mean (SD)** ^5^	1.88 (0.25)	1.85 (0.23)	1.90 (0.26)
**Initial dose intensity, mean (SD)** ^6^	82 (21)	52 (18)	90 (12)
**Average dose intensity, mean (SD)**	80 (18)	54 (14)	87 (13)
**Treatment cycles, median (range)** ^7^	5 (1–24)	5 (1–20)	4 (1–24)

^1^ *DPYD* variants include c.2846A>T, c.1905+1G>A, c.1679T>G, and c.1236G>A; ^2^ includes those with and without biologic agents; ^3^ includes gemcitabine, lapatinib, temozolomide, docetxel, epirubicin, and mitomycin + radiation; ^4^ includes degramount, FEC-D, and FLOT regimens, in addition to mitomycin + radiation; ^5^ body surface area; ^6^ the percentage of ideal dose for each patient given their regimen and body surface area; ^7^ the number of treatment cycles attempted in each patient, though some cycles were discontinued early due to adverse events.

**Table 3:** Severe fluoropyridine-related adverse events during total treatment period.

	Genotype	Grade ≥ 3 Toxicity ^1^ during Total Treatment Period N, (%)	OR (95% CI) (Adjusted)	*p*-Value
	**miR-27a rs895819**			
Total Population	A/A	25 (25)	1.0 (reference)	
	A/G	38 (43)	2.38 (1.26 to 4.57)	0.0079
	G/G	12 (32)	1.49 (0.63 to 3.43)	0.3588
*DPYD* Wildtype	A/A	22 (28)	1.0 (reference)	
	A/G	30 (43)	1.99 (1.00 to 3.99)	0.0507
	G/G	11 (35)	1.44 (0.58 to 3.49)	0.4278
*DPYD* Variant Carriers ^2^	A/A	3 (15)	1.0 (reference)	
	A/G	8 (42)	8.10 (1.16 to 86.21)	0.0497
	G/G	1 (17)	3.87 (0.12 to 90.67)	0.3879
	**miR-27a rs11671784**			
Total Population	C/C	74 (34)	1.0 (reference)	
	C/T	1 (14)	0.33 (0.02 to 2.06)	0.3155
*DPYD* Wildtype	C/C	63 (36)	-	-
	C/T	0 (0)		
*DPYD* Variant Carriers ^2^	C/C	11 (25)	-	-
	C/T	1 (100)		

^1^ Grade ≥ 3 based on the Common Terminology Criteria for Adverse Events (version 5.0); ^2^ *DPYD* variants include c.2846A>T, c.1905+1G>A, c.1679T>G, and c.1236G>A.

**Table 4:** Severe fluoropyridine-related adverse events during cycles 1 and 2.

	Genotype	Grade ≥ 3 Toxicity ^1^ during Cycles 1 and 2 N, (%)	OR (95% CI) (Adjusted)	*p*-Value
	**miR-27a rs895819**			
Total Population	A/A	18 (18)	1.00 (reference)	
	A/G	28 (32)	2.29 (1.14 to 4.71)	0.0219
	G/G	7 (19)	1.05 (0.37 to 2.75)	0.9206
*DPYD* Wildtype	A/A	15 (19)	1.00 (reference)	
	A/G	24 (35)	2.30 (1.09 to 4.97)	0.0310
	G/G	7 (23)	1.21 (0.41 to 3.27)	0.7209
*DPYD* Variant Carrier ^2^	A/A	3 15)	-	-
	A/G	4 (21)		
	G/G	0 (0)		
	**miR-27a rs11671784**			
Total Population	C/C	52 (24)	1.00 (reference)	
	C/T	1 (14)	0.56 (0.03 to 3.54)	0.6029
*DPYD* Wildtype	C/C	46 (26)	-	-
	C/T	0 (0)		
*DPYD* Variant Carrier ^2^	C/C	6 (14)	-	-
	C/T	1 (100)		

^1^ Grade ≥ 3 based on the Common Terminology Criteria for Adverse Events (version 5.0); ^2^ *DPYD* variants include c.2846A>T, c.1905+1G>A, c.1679T>G, and c.1236G>A.

**Table 5:** Number of severe fluoropyridine-related adverse events during the total treatment period.

miR-27a rs895819		Grade ≥ 3 Adverse Events ^1^ during Total Treatment Period					Discontinued Treatment ^3^	Death ^4^
		GI	MS	Cardiac	HFS	Other ^2^		
Total Population	A/A	8 (8)	10 (10)	1 (1)	2 (2)	11 (11)	16 (16)	0 (0)
	A/G	13 (15)	19 (22)	3 (3)	4 (5)	7 (8)	20 (23)	1 (1)
	G/G	3 (8)	5 (14)	1 (3)	0 (0)	6 (16)	6 (16)	0 (0)
*DPYD* Wildtype	A/A	7 (9)	7 (9)	1 (1)	2 (3)	10 (13)	12 (15)	0 (0)
	A/G	9 (13)	16 (23)	3 (3)	2 (3)	6 (9)	14 (20)	1 (1)
	G/G	2 (6)	5 (16)	1 (3)	0 (0)	6 (19)	6 (19)	0 (0)
*DPYD* Variant Carriers ^5^	A/A	1 (5)	3 (15)	0 (0)	0 (0)	1 (5)	4 (20)	0 (0)
	A/G	4 (21)	3 (16)	0 (0)	2 (11)	1 (5)	6 (32)	0 (0)
	G/G	1 (17)	0 (0)	0 (0)	0 (0)	0 (0)	0 (0)	0 (0)

Abbreviations: GI, gastrointestinal; MS, myelosuppression; HFS, hand–foot syndrome; ^1^ grade ≥ 3 based on the Common Terminology Criteria for Adverse Events (version 5.0); ^2^ other grades with ≥ 3 adverse events (including fatigue, infections, neurotoxicity, and laboratory abnormalities); ^3^ the discontinuation of treatment due to fluoropyrimidine-related adverse events of any grade; ^4^ at least one fluoropyrimidine-related adverse event contributed significantly to death; ^5^ *DPYD* variants include c.2846A>T, c.1905+1G>A, c.1679T>G, and c.1236G>A.

**Table 6:** Number of severe fluoropyridine-related adverse events during cycles 1 and 2.

miR-27a rs895819		Grade ≥ 3 Adverse Events ^1^ during Total Treatment Period					Discontinued Treatment ^3^	Death ^4^
		GI	MS	Cardiac	HFS	Other ^2^		
Total Population	A/A	8 (8)	6 (6)	1 (1)	1 (1)	8 (8)	9 (9)	0 (0)
	A/G	11 (13)	16 (18)	2 (2)	3 (3)	4 (5)	11 (13)	0 (0)
	G/G	2 (5)	4 (11)	1 (3)	0 (0)	2 (5)	4 (11)	0 (0)
*DPYD* Wildtype	A/A	7 (8)	3 (4)	1 (1)	1 (1)	7 (9)	5 (6)	0 (0)
	A/G	9 (13)	14 (20)	2 (3)	2 (3)	3 (4)	10 (14)	0 (0)
	G/G	2 (6)	4 (13)	1 (3)	0 (0)	2 (6)	4 (13)	0 (0)
*DPYD* Variant Carriers ^5^	A/A	1 (5)	3 (15)	0 (0)	0 (0)	1 (5)	4 (20)	0 (0)
	A/G	2 (11)	2 (11)	0 (0)	1 (5)	1 (5)	1 (5)	0 (0)
	G/G	0 (0)	0 (0)	0 (0)	0 (0)	0 (0)	0 (0)	0 (0)

Abbreviations: GI, gastrointestinal; MS, myelosuppression; HFS, hand–foot syndrome; ^1^ grade ≥ 3 based on the Common Terminology Criteria for Adverse Events (version 5.0); ^2^ other grade with ≥ 3 adverse events (including fatigue, infections, neurotoxicity, and laboratory abnormalities); ^3^ the discontinuation of treatment due to fluoropyrimidine-related adverse events of any grade; ^4^ at least one fluoropyrimidine-related adverse event contributed significantly to death; ^5^ *DPYD* variants include c.2846A>T, c.1905+1G>A, c.1679T>G, and c.1236G>A.

## Data Availability

The datasets generated and/or analyzed during the current study are available from the corresponding author on reasonable request.

## Supplementary Materials

The supporting information can be downloaded at https://www.mdpi.com/article/10.3390/ijms241713284/s1.

## References

[1] Wigle T.J., Tsvetkova E.V., Welch S.A., Kim R.B. (2019). Fluorouracil-Based Chemotherapy: Mini Review and Case Report. Pharmaceutics.

[2] Amstutz U., Henricks L.M., Offer S.M., Barbarino J., Schellens J.H.M., Swen J.J., Klein T.E., McLeod H.L., Caudle K.E., Diasio R.B. (2018). Clinical Pharmacogenetics Implementation Consortium (CPIC) Guideline for Dihydropyrimidine Dehydrogenase Genotype and Fluoropyrimidine Dosing: 2017 Update. Clin. Pharmacol. Ther..

[3] Bartel D.P. (2004). MicroRNAs: Genomics, biogenesis, mechanism, and function. Cell.

[4] Komatsu S., Kitai H., Suzuki H.I. (2023). Network Regulation of microRNA Biogenesis and Target Interaction. Cells.

[5] Iorio M.V., Croce C.M. (2017). MicroRNA dysregulation in cancer: Diagnostics, monitoring and therapeutics. A comprehensive review. EMBO Mol. Med..

[6] He B., Zhao Z., Cai Q., Zhang Y., Zhang P., Shi S., Xie H., Peng X., Yin W., Tao Y. (2020). miRNA-based biomarkers, therapies, and resistance in Cancer. Int. J. Biol. Sci..

[7] Ali Syeda Z., Langden S.S.S., Munkhzul C., Lee M., Song S.J. (2020). Regulatory Mechanism of MicroRNA Expression in Cancer. Int. J. Mol. Sci..

[8] Hezova R., Kovarikova A., Bienertova-Vasku J., Sachlova M., Redova M., Vasku A., Svoboda M., Radova L., Kiss I., Vyzula R. (2012). Evaluation of SNPs in miR-196-a2, miR-27a and miR-146a as risk factors of colorectal cancer. World J. Gastroenterol..

[9] Kupcinskas J., Bruzaite I., Juzenas S., Gyvyte U., Jonaitis L., Kiudelis G., Skieceviciene J., Leja M., Pauzas H., Tamelis A. (2014). Lack of association between miR-27a, miR-146a, miR-196a-2, miR-492 and miR-608 gene polymorphisms and colorectal cancer. Sci. Rep..

[10] Wang Z., Sun X., Wang Y., Liu X., Xuan Y., Hu S. (2014). Association between miR-27a genetic variants and susceptibility to colorectal cancer. Diagn. Pathol..

[11] Bian Q., Chen J.J., Gu J.P., Xu J. (2015). Association between pre-miR-27a functional polymorphism and risk of colorectal cancer in north Chinese Han population. Onco Targets Ther..

[12] Jiang Y., Lin D.H., Xu J.P., Chen W.X., Zheng S.J., Song L. (2016). Genotype GG of rs895819 Functional Polymorphism Within miR-27a Might Increase Genetic Susceptibility to Colorectal Cancer in Han Chinese Population. J. Clin. Lab. Anal..

[13] Cao Y., Hu J., Fang Y., Chen Q., Li H. (2014). Association between a functional variant in microRNA-27a and susceptibility to colorectal cancer in a Chinese Han population. Genet. Mol. Res..

[14] Feng Y., Duan F., Song C., Zhao X., Dai L., Cui S. (2016). Systematic evaluation of cancer risk associated with rs2292832 in miR-149 and rs895819 in miR-27a: A comprehensive and updated meta-analysis. Oncotarget.

[15] Liu F., Dear K., Huang L., Liu L., Shi Y., Nie S., Liu Y., Lu Y., Xiang H. (2016). Association between microRNA-27a rs895819 polymorphism and risk of colorectal cancer: A meta-analysis. Cancer Genet..

[16] Chen M., Fang W., Wu X., Bian S., Chen G., Lu L., Weng Y. (2017). Distinct effects of rs895819 on risk of different cancers: An update meta-analysis. Oncotarget.

[17] Dai J., Chen Y., Gong Y., Gu D., Chen J. (2020). Association of microRNA-27a rs895819 polymorphism with the risk of cancer: An updated meta-analysis. Gene.

[18] Yang X., Li X., Hao X., Tian W., Zhou B. (2020). Association of miR-27a polymorphism with the risk of digestive system cancers. Pathol. Res. Pract..

[19] Alidoust M., Hamzehzadeh L., Rivandi M., Pasdar A. (2018). Polymorphisms in non-coding RNAs and risk of colorectal cancer: A systematic review and meta-analysis. Crit. Rev. Oncol. Hematol..

[20] Pan X.M., Xiao X., Qin H.J., Zhang Z., Li Z.H., Gao L.B., Jia J. (2016). MicroRNA variants and colorectal cancer risk: A meta-analysis. Genet. Mol. Res..

[21] Yuan L., Zhang T.T., Ren Y. (2015). miR-27a rs895819 polymorphism and risk of cancer in Chinese population: A meta-analysis. J. Evid. Based Med..

[22] Rong G.Q., Zhang X.M., Chen B., Yang X.D., Wu H.R., Gong W. (2017). MicroRNA gene polymorphisms and the risk of colorectal cancer. Oncol. Lett..

[23] Ma X.P., Zhang T., Peng B., Yu L., Jiang D.K. (2013). Association between microRNA polymorphisms and cancer risk based on the findings of 66 case-control studies. PLoS ONE.

[24] Shankaran Z.S., Walter C.E.J., Prakash N., Ramachandiran K., Priya Doss C.G., Johnson T. (2020). Investigating the role of microRNA-27a gene polymorphisms and its interactive effect with risk factors in gastrointestinal cancers. Heliyon.

[25] Park J.H., Jeong G.H., Lee K.S., Lee K.H., Suh J.S., Eisenhut M., van der Vliet H.J., Kronbichler A., Stubbs B., Solmi M. (2020). Genetic variations in MicroRNA genes and cancer risk: A field synopsis and meta-analysis. Eur. J. Clin. Investig..

[26] Deac A.L., Burz C.C., Militaru C., Bocșan I.C., Pop R.M., Achimaș-Cadariu P., Buzoianu A.D. (2021). Role of microRNAs in fluoropyrimidine-related toxicity: What we know. Eur. Rev. Med. Pharmacol. Sci..

[27] Offer S.M., Butterfield G.L., Jerde C.R., Fossum C.C., Wegner N.J., Diasio R.B. (2014). microRNAs miR-27a and miR-27b directly regulate liver dihydropyrimidine dehydrogenase expression through two conserved binding sites. Mol. Cancer Ther..

[28] Hirota T., Date Y., Nishibatake Y., Takane H., Fukuoka Y., Taniguchi Y., Burioka N., Shimizu E., Nakamura H., Otsubo K. (2012). Dihydropyrimidine dehydrogenase (DPD) expression is negatively regulated by certain microRNAs in human lung tissues. Lung Cancer.

[29] Falvella F.S., Cheli S., Martinetti A., Mazzali C., Iacovelli R., Maggi C., Gariboldi M., Pierotti M.A., Di Bartolomeo M., Sottotetti E. (2015). DPD and UGT1A1 deficiency in colorectal cancer patients receiving triplet chemotherapy with fluoropyrimidines, oxaliplatin and irinotecan. Br. J. Clin. Pharmacol..

[30] Amstutz U., Offer S.M., Sistonen J., Joerger M., Diasio R.B., Largiadèr C.R. (2015). Polymorphisms in MIR27A Associated with Early-Onset Toxicity in Fluoropyrimidine-Based Chemotherapy. Clin. Cancer Res..

[31] Meulendijks D., Henricks L.M., Amstutz U., Froehlich T.K., Largiadèr C.R., Beijnen J.H., de Boer A., Deenen M.J., Cats A., Schellens J.H. (2016). Rs895819 in MIR27A improves the predictive value of DPYD variants to identify patients at risk of severe fluoropyrimidine-associated toxicity. Int. J. Cancer.

[32] Deng Y., Bai H., Hu H. (2015). rs11671784 G/A variation in miR-27a decreases chemo-sensitivity of bladder cancer by decreasing miR-27a and increasing the target RUNX-1 expression. Biochem. Biophys. Res. Commun..

[33] Yang Q., Jie Z., Ye S., Li Z., Han Z., Wu J., Yang C., Jiang Y. (2014). Genetic variations in miR-27a gene decrease mature miR-27a level and reduce gastric cancer susceptibility. Oncogene.

[34] Song B., Yan G., Hao H., Yang B. (2014). rs11671784 G/A and rs895819 A/G polymorphisms inversely affect gastric cancer susceptibility and miR-27a expression in a Chinese population. Med. Sci. Monit..

[35] Yang Y., Lu W., Ning M., Zhou X., Wan X., Mi Q., Yang X., Zhang D., Zhang Y., Jiang B. (2022). A functional SNP rs895819 on pre-miR-27a is associated with bipolar disorder by targeting NCAM1. Commun. Biol..

[36] Tang W., Xu H., Ma D., Ma R., Wu J., Yu X., Feng J., Liu Q. (2020). Pre-miR-27a rs895819 polymorphism and risk of diffuse large B-cell lymphoma. J. Clin. Lab. Anal..

[37] Takuse Y., Watanabe M., Inoue N., Ozaki R., Ohtsu H., Saeki M., Katsumata Y., Hidaka Y., Iwatani Y. (2017). Association of IL-10-Regulating MicroRNAs in Peripheral Blood Mononuclear Cells with the Pathogenesis of Autoimmune Thyroid Disease. Immunol. Investig..

[38] Radanova M., Levkova M., Mihaylova G., Manev R., Maneva M., Hadgiev R., Conev N., Donev I. (2022). Single Nucleotide Polymorphisms in microRNA Genes and Colorectal Cancer Risk and Prognosis. Biomedicines.

[39] Wang X., Xing Y., Wang Y., Du Z., Zhang C., Gao J. (2023). Association of microRNA gene polymorphisms with recurrent spontaneous abortion: An updated meta-analysis. Exp. Ther. Med..

[40] Gholami M., Asgarbeik S., Razi F., Esfahani E.N., Zoughi M., Vahidi A., Larijani B., Amoli M.M. (2020). Association of microRNA gene polymorphisms with Type 2 diabetes mellitus: A systematic review and meta-analysis. J. Res. Med. Sci..

[41] Medwid S., Li M.M.J., Knauer M.J., Lin K., Mansell S.E., Schmerk C.L., Zhu C., Griffin K.E., Yousif M.D., Dresser G.K. (2019). Fexofenadine and Rosuvastatin Pharmacokinetics in Mice with Targeted Disruption of Organic Anion Transporting Polypeptide 2B1. Drug Metab. Dispos..

[42] Wigle T.J., Povitz B.L., Medwid S., Teft W.A., Legan R.M., Lenehan J., Nevison S., Panuganty V., Keller D., Mailloux J. (2021). Impact of pretreatment dihydropyrimidine dehydrogenase genotype-guided fluoropyrimidine dosing on chemotherapy associated adverse events. Clin. Transl. Sci..

[43] Yang R., Burwinkel B. (2012). A bias in genotyping the miR-27a rs895819 and rs11671784 variants. Breast Cancer Res. Treat..

