# Supplementary material for: Genetic Variation in miR-27a Is Associated with Fluoropyrimidine-Associated Toxicity in Patients with Dihydropyrimidine Dehydrogenase Variants after Genotype-Guided Dose Reduction
Source: Int J Mol Sci. 2023 Aug 27;24(17):13284. doi: 10.3390/ijms241713284 (PMC10487873; doi:10.3390/ijms241713284)
Supplement: Supplementary file 1 [file ijms-24-13284-s001.zip › ijms-2561547-supplementary.pdf]

Supplemental Figures

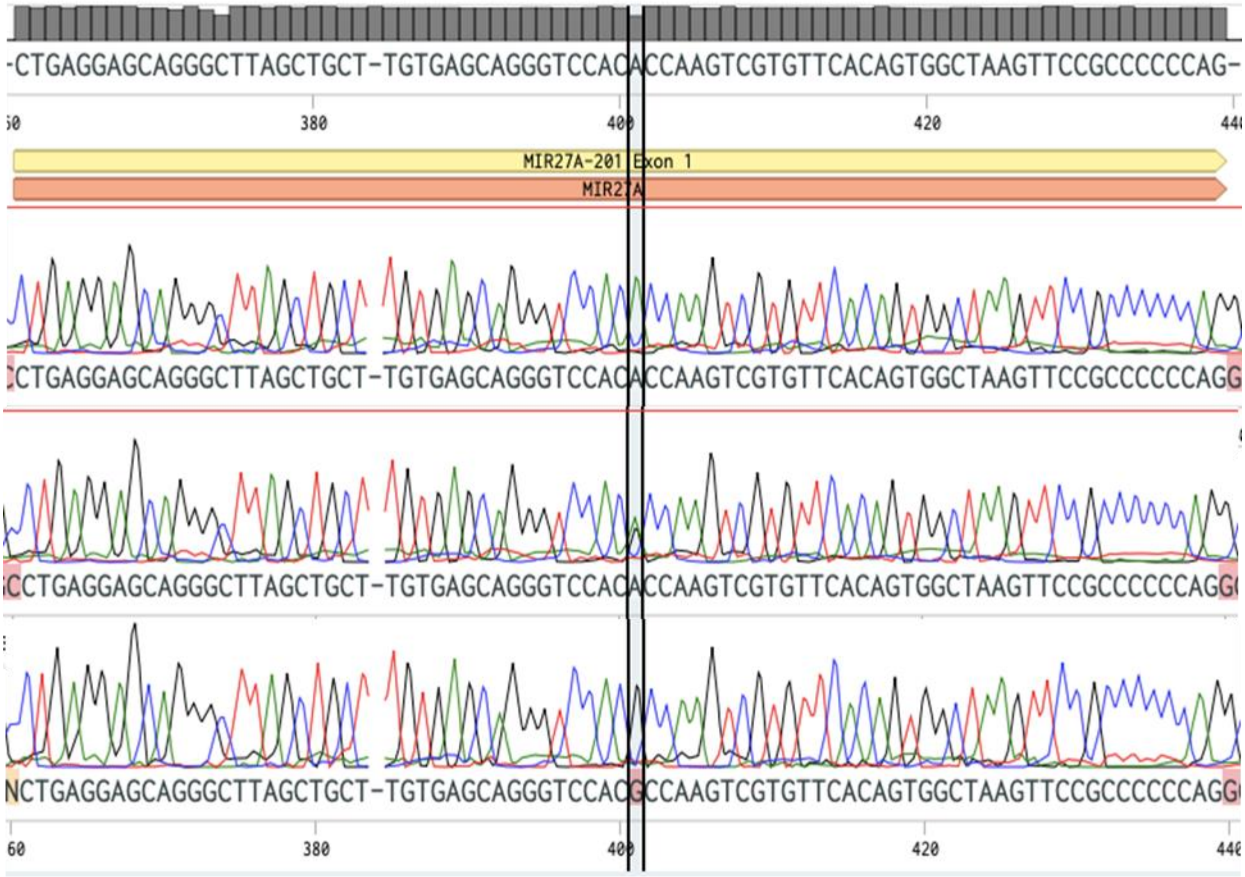

Figure S1. Sanger sequencing for miR27A. Highlighted is rs895819, with the top panel showing a homozygous wildtype, middle heterozygote and bottom homozygous variant.

Table S1. Severe fluoropyridine related adverse events during total treatment period.

	**Genotype**	**Grade ≥ 3 Toxicity[1] during total treatment period N, (%)**	**OR (95% CI) (adjusted)**	**P-value**
Total Treatment Period	Wildtype<br>*DPYD* Carriers[2]	63 (35)<br>12 (27)	1.0 (reference)<br>0.67 (0.31 to 1.36)	 0.2792
Cycles 1 & 2	Wildtype<br>*DPYD* Carriers[2]	46 (26)<br>7 (16)	1.00 (reference)<br>0.53 (0.20 to 1.21)	 0.1536

[1]Grade ≥ 3 by Common Terminology Criteria for Adverse Events version 5.0; [2]*DPYD* variants include c.2846A>T, c.1905+1G>A, c.1679T>G and c.1236G>A.
